# The protective effects of *Allium ampeloprasum *Subsp* Iranicum *on cyclophosphamide-induced immunosuppression in NMRI Mice: A promising natural immunomodulator 

**DOI:** 10.22038/AJP.2024.24595

**Published:** 2024

**Authors:** Fatemeh Abedi, Bahareh Sadat Yousefsani, Kobra Shirani

**Affiliations:** 1 *Department of Toxicology, Faculty of Medicine Sciences, Tarbiat Modares University, Tehran, Iran*; 2 *Research Institute for Islamic and Complementary Medicine, Iran University of Medical Sciences, Tehran, Iran*; 3 *School of Persian Medicine, Iran University of Medical Sciences, Tehran, Iran*

**Keywords:** Cyclophosphamide, Allium ampeloprasum, Subsp Iranicum, Immunomodulatory

## Abstract

**Objective:**

Cyclophosphamide (CTX) is an effective anticancer drug, but toxic effects against normal human tissues are its dose-limiting drawback. The aim of this research was to investigate the *in vivo* immunomodulatory activities of *Allium ampeloprasum *Subsp* Iranicum *against CTX-induced toxicity in mice.

**Materials and Methods:**

Extract of the whole plant of *A. ampeloprasum *Subsp* Iranicum *was obtained using the maceration technique, and its total phenolic and flavonoid contents were quantified through spectrophotometric analysis. Mice received daily oral administration (PO) of the extract (150 mg/kg) for a duration of 14 days, either as a standalone treatment or in conjunction with an intraperitoneal (IP) injection of 20 mg/kg CTX. The effects of the extract on body weight, spleen weight, white blood cell (WBC), serum antibody titer hemagglutination (HA), delayed-type hypersensitivity response (DTH), lymphocyte proliferation, cytokine production, and histopathological examinations were evaluated.

**Results:**

*A. ampeloprasum *Subsp* Iranicum *restored several parameters including spleen weight (p<0.001), WBC (p<0.001), lymphocytes (p<0.05), and monocytes (p<0.01), HA titer (p<0.05), and DTH response (p<0.01). *A. ampeloprasum *Subsp* Iranicum *notably stimulated lymphocyte proliferation to PHA (p<0.01) and LPS (p<0.05) and recovered interferon (IFN)-γ (p<0.001) and interleukin (IL)-4 (p<0.001) levels in the immunosuppressed mice. Also, CTX-induced histopathological changes were reversed by *A. ampeloprasum *Subsp* Iranicum.*

**Conclusion:**

Analyses revealed *A. ampeloprasum *Subsp* Iranicum *could regulate immunity and increase host immune responses. The observed strengthening effect can be attributed to the high amount of flavonoids and dipropyl trisulfide compounds present in *A. ampeloprasum *Subsp* Iranicum*.

## Introduction

The immune system is a complex web of mechanisms that play an important role in protecting the internal environment of the body against disease agents (Nicholson, 2016). In the immune system, two different types of reactions to pathogens and invading agents are observed: innate responses and acquired responses (Delves and Roitt, 2000). Immunodeficiency disorders may result from a primary genetic defect that can affect the function of the innate or acquired immune system, through inhibition of immune cells or pathways, or may result from a secondary immunodeficiency such as environmental conditions, viral or bacterial infections, malnutrition, autoimmunity or treatment with drugs that suppress the immune system (Marshall et al., 2018). In immunodeficiency, the ability of the immune system to fight infectious diseases is compromised or completely absent and people whose immune function is impaired show a greater susceptibility to infection and morbidity and even mortality (Descotes, 2005; Gleichmann et al., 1989).

Cyclophosphamide (CTX) is mainly used to control and treat cancer and autoimmune diseases, and is prescribed before transplantation to prevent graft rejection and transplant complications in the host. This drug is a type of nitrogen mustard drug that exerts its effects through DNA alkylation, inhibiting protein synthesis through DNA and RNA cross-linking, which eventually leads to programmed cell death. Nevertheless, the primary limitation in CTX therapy stems from its cytotoxic effects on healthy human tissues, which serve as the main factor restricting dosage and consequently impacting treatment protocols and overall quality of life. The prominent drawback associated with the utilization of synthetic immunomodulatory agents resides in their adverse reactions, including neutropenia, anorexia, and proteinuria (Ahlmann and Hempel, 2016; Emadi et al., 2009). 

Medicinal plants have been used in health care since ancient times (Bayan et al., 2014). Due to the fact that some plants have immunomodulatory properties and low toxicity, they can be a suitable alternative to conventional chemical drugs (Abtahi Froushani et al., 2015; Shirani et al., 2022). Among these medicinal plants, *Allium* genus could ameliorate chemotherapy symptoms by stimulating both innate and adaptive immunity. *Allium* genus, belonging to the family Amaryllidaceae, is recognized as one of the most extensive monocotyledonous genera. This botanical group comprises diverse culinary herbs such as garlic, onion, shallot, leek, chives, and scallions. *Allium* genus has been used in the treatment of a diverse array of pathological conditions due to its antibacterial, antifungal, blood sugar lowering, blood pressure lowering, blood cholesterol lowering, and anti-atherosclerotic properties (Lanzotti et al., 2014; Ruhee et al., 2020; Teshika et al., 2019).

*Allium ampeloprasum *Subsp* Iranicum *is a fascinating plant that delights our taste buds with its onion-like flavor, making it a popular choice for raw consumption and culinary experimentation. With its ten long flower stalks and broad leaves, this plant displays a unique tuberous structure. This remarkable plant is a rich source of starch, dietary fibers, carbohydrates, proteins, minerals, and an array of bioactive phytochemicals. It is like a treasure trove of goodness! Not to mention, it boasts an impressive lineup of antioxidants and important vitamins such as A, B, and C. Drawing inspiration from other members of the* Allium *genus*, A. ampeloprasum *Subsp* Iranicum *exhibits a wide range of pharmacological activities. It has been found to possess remarkable potential as an antidiabetic, anti-inflammatory, hypolipidaemic, anticarcinogenic, and antimicrobial agent. Additionally, its powerful free radical scavenging abilities make it an excellent choice for combatting oxidative stress. This plant also shows promising effects as an antihelmintic, diuretic, and antihypertensive effects, and possesses digestive properties (Mehdizadeh et al., 2021; Fatoorechi et al., 2016; Shiehzadeh et al., 2022).

The aim of this research was to investigate the in* vivo* immunomodulatory activities of a hydroalcoholic extract of *A. ampeloprasum *Subsp* Iranicum* against CTX-induced immunosuppression in NMRI mice by investigating immune organ index, white blood cell count, serum antibody titer hemagglutination (HA), delayed-type hypersensitivity (DTH), splenocyte proliferation, NK cell activity, cytokine production, and histopathological examination.

## Materials

### Plant material

*A. ampeloprasum *Subsp* Iranicum* was gathered during the spring of 2023 from the scenic Chaharmahal VA Bakhtiari Mountains in Iran. A team of skilled botanists from the Islamic and Complementary Medicine department, Iran University of Medical Sciences, Tehran, Iran, precisely identified the specimen.

### Plant extraction

All parts of *A. ampeloprasum *Subsp* Iranicum *were dried in the shade and ground into powder by a blender. The whole plant powder (10 g) was extracted using the maceration method with 100 ml of 80% aqueous ethanol. The solvent was filtered every 24 hr and fresh solvent was added for a total duration of 3 days. All the extracts were combined and underwent drying using both a rotary evaporator and freeze dryer at a temperature kept below 40℃ (Yousefsani et al., 2022).

### Measurement of total phenolic compounds

The spectrophotometric determination of the total phenolic content was conducted using the Folin-Ciocalteu method, with gallic acid as the standard described previously (Fattahi et al., 2014).

### Measurement of total flavonoid compounds

The total flavonoid content was measured using a colorimetric assay described previously (Chang et al., 2002).

### Animals

The mice utilized in the study were acquired from the Faculty of Medical Sciences, Tarbiat Modares University. These mice, obtained from the Naval Medical Research Institute (NMRI), were male, aged 6 to 8 weeks, with a weight ranging from 19 to 21 g. Before any experiments began, the mice were housed in polystyrene cages accommodating five mice per cage. They resided in the laboratory environment for a duration of 1 week, adhering to standard conditions. These standard conditions entailed maintaining a temperature of 20–22°C, a relative humidity of 35%, and a light-dark cycle of 12 hr each. Throughout this observational period, the mice had unrestricted access to both food and water, provided *ad libitum*. All animal experiments conducted during this study obtained approval from the ethical committee of Tarbiat Modares University, with the assigned approval number IR.MODARES.AEC.1401.014.

### Doses and exposure schedules

A total of sixty mice were grouped randomly with each group comprising twenty mice (n=20). Each of these groups was further subdivided into four subsets, where each subset consisted of five mice (n=5).

(i) In the first subset, the mice received 150 mg/kg *A. ampeloprasum *Subsp* Iranicum (*PO) for a duration of 14 days. (ii) The second subset of mice received 150 mg/kg *A. ampeloprasum *Subsp* Iranicum *(14 days) (PO) combined with IP injection of 20 mg/kg CTX (5 days). (iii) The third subset underwent IP injection of 20 mg/kg CTX for a period of 5 days, and this served as the positive control group. (iv) The fourth subset of mice received oral administration of normal saline for 14 days, representing the negative control group (Riahi-Zanjani et al., 2015; Shiehzadeh et al., 2022).

### Body and spleen weight

In order to evaluate the weight of the mice, their weight was measured on the first day before administering the first dose and on the 14th day, 2 hr after the last dose. After measuring their weight, the animals were anesthetized using ketamine at a dosage of 100 mg/kg and xylazine at 10 mg/kg, then they were sacrificed by decapitation, and the weight of their spleen was recorded.

### White blood cell count

On the 14th day, 2 hr after administering the last dose, 0.2 ml of blood was collected from the Retro-orbital plexus into tubes stained with EDTA-K2. Total WBC counts, lymphocytes, **neutrophil** and monocytes were determined.

### Preparation of single-cell suspension

On the 14th day, two hours after the final dose administration, the mice were euthanized by severing the spinal cord. Subsequently, the spleen was aseptically extracted from the mice. Using a needle and syringe, the spleen was processed into a cell suspension using RPMI1640 medium. To separate any remaining spleen tissue debris, the cell suspension was passed through a cell strainer. The cells were then centrifuged at 224 g and 4°C for 10 min. The supernatant was discarded, and the remaining sediment was resuspended in approximately 3 ml of red blood cell (RBC) lysis solution for 3 min at room temperature. Afterward, the cells were centrifuged again at 224 g and 4°C for 5 min. The resulting supernatant was discarded. The cells were washed three times using RPMI1640 culture medium. To determine the percentage of cell viability, a cell suspension was prepared in 5% phosphate buffer saline (PBS) and mixed with 0.4% trypan blue. By examining the cells under an optical microscope, the percentage of cell viability was determined (Riahi-Zanjani et al., 2015).

### HA test

To perform the experiment, 1 mL of washed sheep red blood cells (SRBCs) diluted using Freund's complete adjuvant until reaching a dilution of 5×10^8^. On the 11th day, 5×10^8 ^SRBCs was administered to the mice via IP injection. At the end of experiment, after providing sera from collected blood samples, aliquots (50 µl) of two-fold dilutions of the sera (in PBS) . Then, we set up multiple test tubes with serial dilutions of the serum. In each test tube, we mixed 25 µl of serum with 25 µl of sheep red blood cells (SRBC) suspension in phosphate buffer. To observe the agglutination reaction, the tubes were incubated at 37°C for 1 hr. The antibody titer was determined by recording the highest dilution at which agglutination was observed (Riahi-Zanjani et al., 2015).

### DTH response

The DTH test was conducted following the method by Fararjeh et al. with some modifications. The preparation of RBC followed a similar procedure as described previously, but with a variation. We continued the dilution process using Freund's complete adjuvant until reaching a dilution of 1×10^9^. On the 11th day, the animals were IP sensitized with 1×10^9^ SRBCs. Then, on the 14th day, a booster dose equivalent to 1×10^9^ SRBCs was injected into the left hind footpad. To control non-specific swelling, a comparable volume of Freund's incomplete adjuvant was injected into the right hind footpad. After 24 and 48 hr, we measured the increase in volume in both paws using a Vernier caliper (Mitutoyo, Kawasaki, Japan). By calculating the difference between the volumes of the left and right hind footpads, we obtained data on the specific response and the impact of non-specific swelling. (Zimecki and Kruzel, 2000).

### Lymphocyte proliferation

The 100 µl aliquots of the splenocytes were placed into wells of a 96-well microtiter plate. To promote cell attachment and stimulation, we did not add any mitogen, PHA, or LPS to the wells, maintaining a final concentration of 5 and 1 μg/ml. The plate was then placed in a controlled incubator at 37°C with 5% CO_2_ for 48 hr. This incubation period allowed the cells to attach to the substrate and induce the desired response. Afterwards, the MTT assay was employed to determine the cells' proliferation. To calculate the proliferation index (PI), we divided the absorbance value of the stimulated cells by the absorbance value of the unstimulated cells. This index provides valuable insights into the relative proliferation rates between the two conditions (Shirani et al., 2021).

### Production of cytokines

To measure the levels of two important cytokines, IFN-γ and IL-4 in the collected supernatants, we utilized commercially available ELISA kits and followed the instructions provided by the manufacturer (Shirani et al., 2021).

### Histopathological examinations

Spleen was collected from each mouse and fixed in 10% formalin. Following mounting, 5-μm thick sections of these tissues were stained with hematoxylin and eosin (H&E) and analyzed via light microscopy. 

### Statistical analysis

The data are expressed as the mean±SD and were analyzed using a one-way ANOVA, followed by Tukey's multiple comparison test with the utilization of PRISM, version 6.00 (GraphPad Software Inc., San Diego, CA, USA). A statistical significance of p<0.05 was deemed as indicating a notable difference.

## Results

### Amount of total phenolic compounds in the extract

The total phenolic content of the extract was 197.85 mg/l g of dry extract.

### Amount of total flavonoid compounds in the extract

The total flavonoid compound of the extract was 334/66 mg/1 g of dry extract.

### Effect on mouse body weight

**Figure 1:**
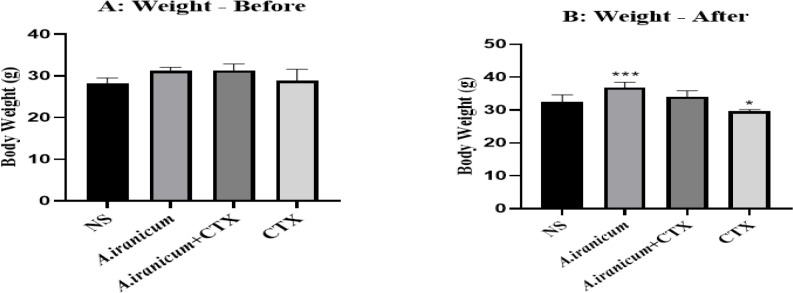
The effects of *A. ampeloprasum *Subsp* Iranicum* extract on mice body weight before (A) and after (B) treatment. Data are expressed as the mean±SD (n=5). *p<0.05 and ***p<0.001 compared to control group (NS). Data were analyzed through one-way ANOVA coupled with Tukey-Kramer multiple comparisons test. NS (Normal Saline), CTX (Cyclophosphamide).

The average body weight showed a significant increase in mice that received *A. ampeloprasum *Subsp* Iranicum * plant extract compared to the normal saline group (p<0.001) (Figure 1A). In addition, in mice that received CTX, a significant decrease was observed compared to the normal saline group (p<0.05) (Figure 1B).

**Figure 2:**
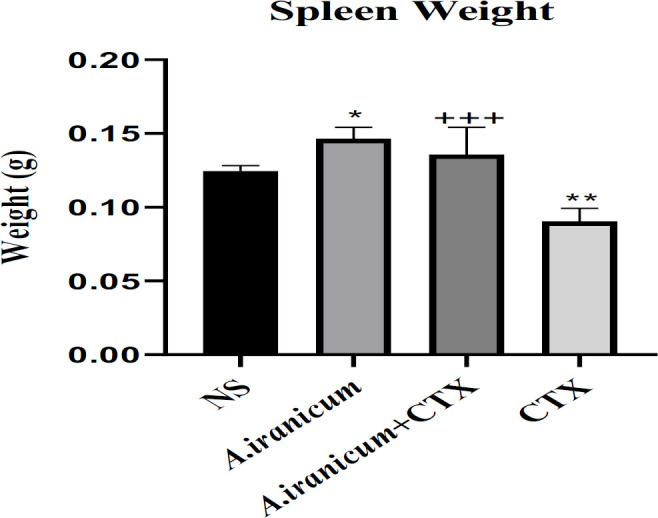
The effects of *A. ampeloprasum *Subsp* Iranicum* extract on spleen weight in CTX exposed mice. Data are expressed as the mean±SD (n=5). *p<0.05 and ***p<0.001 compared to control group (NS), +++p<0.001 compared to CTX group. Data were analyzed through one-way ANOVA coupled with Tukey-Kramer multiple comparisons test. NS (Normal Saline) and CTX (Cyclophosphamide).

As shown in Figure 2, spleen weight in the group that received *A. ampeloprasum *Subsp* Iranicum* extract alone showed a significant enhance compared to the normal saline group (p<0.05). On the other hand, a meaningfully decrease was seen in the CTX group compared to the normal saline group (p<0.01). In addition, *A. ampeloprasum *Subsp* Iranicum* extract (150 mg/kg) markedly enhanced spleen weight compared to CTX group (p<0.001) (Figure 2). 

### White blood cell counts

The administration of CTX (50 mg/kg) resulted in a significant decrease in the number of WBC, lymphocytes, monocytes, and; when comparing to the control group. However, introducing *A. ampeloprasum *Subsp* Iranicum* at a dose of 150 mg/kg demonstrated a notable increase in white blood cell count. Moreover, the extract from this plant exhibited the ability to alleviate the reduction in white blood cells, lymphocytes, and monocytes induced by CTX, as summarized in Table 1.

### Spleen cell number

**Figure 3:**
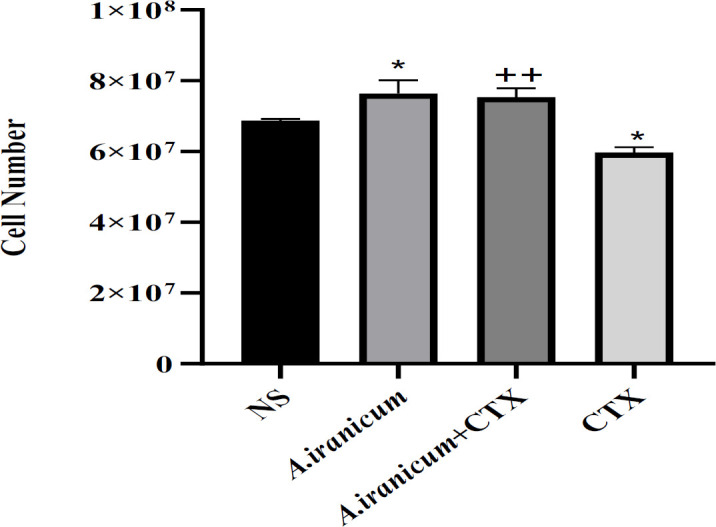
The effects of *A. ampeloprasum *Subsp* Iranicum* extract on spleen cell number in CTX-exposed mice. Data are expressed as the mean±SD (n=5 animals). *p<0.05 compared to control group (NS), ++p<0.01 compared to CTX group. Data were analyzed through one-way ANOVA coupled with Tukey-Kramer multiple comparisons test. NS (Normal Saline) and CTX (Cyclophosphamide).

As observed in Figure 3, in mice that received *A. ampeloprasum *Subsp* Iranicum* extract, there was a significant increase in spleen cells compared to the normal saline group (p<0.05) whereas administration of CTX markedly decreased it. The decreased spleen cell numbers in the CTX group were reversed by *A. ampeloprasum *Subsp* Iranicum * (p<0.01) compared to the CTX only exposed group**. **

### Serum antibody titer

**Figure 4:**
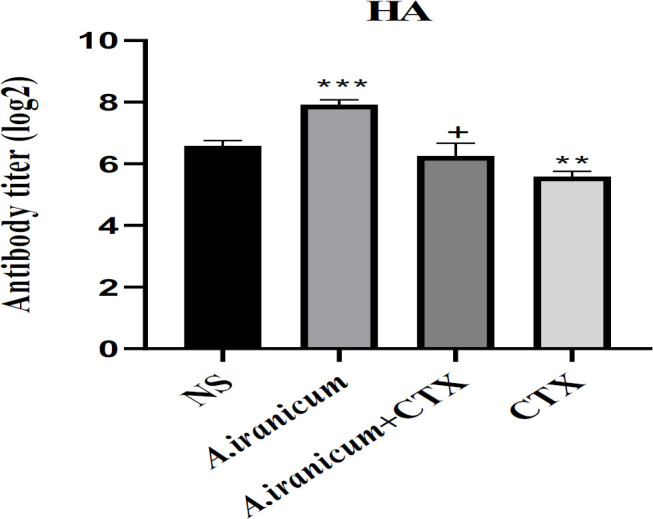
The effects of *A. ampeloprasum *Subsp* Iranicum* extract on hemagglutination (HA) titer in CTX-exposed mice. Data are expressed as the mean±SD (n=5). **p<0.01, ***p<0.001 compared to control group (NS), +p<0.05 compared to CTX group. Data were analyzed through one-way ANOVA coupled with Tukey-Kramer multiple comparisons test. NS (Normal Saline) and CTX (Cyclophosphamide).

Results of the hemagglutination (HA) titer presented an obvious decline in antibody production in CTX - treated mice compared to the normal saline group (p<0.001). In addition, a significant increase in antibody production was observed in the group that received *A. ampeloprasum *Subsp* Iranicum *extract compared to the normal saline group (p<0.01). Also, the ability to produce antibodies in the mice that received the *A. ampeloprasum *Subsp* Iranicum* extract + CTX was significantly higher than the CTX group (p<0.05) (Figure 4). 

### DTH response

**Figure 5:**
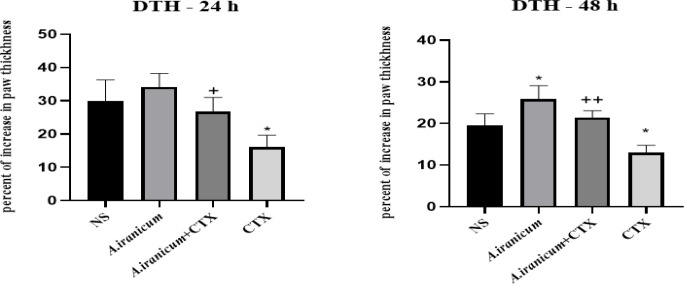
The effects of *A. ampeloprasum *Subsp* Iranicum extract* on delayed-type hypersensitivity (DTH) response after 24 (A) and 48 hr (B) in CTX-exposed mice. Data are expressed as mean±SD (n=5). *p<0.05 compared to control group (NS), + p<0.05 and ++p<0.01 compared to CTX group. Data were analyzed through one-way ANOVA coupled with Tukey-Kramer multiple comparisons test. NS (Normal Saline) and CTX (Cyclophosphamide).

As depicted in Figure 5A, a statistically significant decrease was seen in the CTX group compared to the normal saline group after 24 hr (p<0.05). Furthermore, there was a significant increase in the DTH response in the *A ampeloprasum *Subsp* Iranicum *extract + CTX group when compared to the CTX group. After 48 hr (Figure 5B), there was a significant elevation in the DTH response in the groups that received *A. ampeloprasum *Subsp* Iranicum *extract compared to the control group (p<0.05) (Figure 5B).

### Lymphocyte proliferation

**Figure 6:**
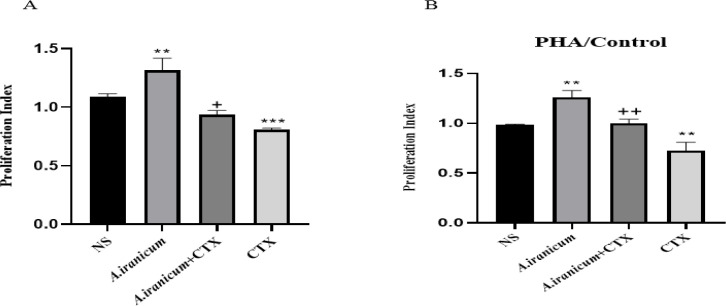
The effects of exposure to *A. ampeloprasum *Subsp* Iranicum on* lymphocyte proliferation response to LPS (A) and PHA (B) in CTX-exposed mice. Data are expressed as mean±SD (n=5). *p<0.05 compared to control group (NS), +p<0.05 and ++p<0.01 compared to CTX group. Data were analyzed through one-way ANOVA coupled with Tukey-Kramer multiple comparisons test. NS (Normal Saline), CTX (Cyclophosphamide).

As presented in Figure (6), *A. ampeloprasum *Subsp* Iranicum* extract remarkably increased cell proliferation in response to PHA and LPS, compared to the normal saline group. On the contrary, a significant decline was observed in the CTX group compared to the normal saline group. 

In addition, *A. ampeloprasum *Subsp* Iranicum *administration significantly attenuated CTX-induced cell proliferation suppression compared to the CTX group.

### Effect on the secretion IFN-γ and IL-4

Mice exposed to CTX exhibited significantly reduced concentrations of IFN-𝛾 and IL-4. However, according to Table 2, the administration of *A. ampeloprasum *Subsp* Iranicum *restored the levels of IFN-𝛾 and IL-4 in the immunosuppressed mice.

### Histopathology

**Figure 7:**
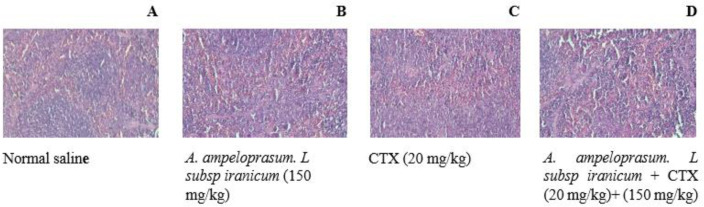
The effects of *A. ampeloprasum *Subsp* Iranicum* *extract* on spleen histopathology after 24 hr in mice. Data are expressed as the mean±SD (n=5). NS (Normal Saline) and CTX (Cyclophosphamide).

The spleen was evaluated for white pulp atrophy (or hyperplasia), red pulp: white pulp (Shirani et al., 2018). As observed in Figure 7A, there were no changes in the normal saline group. Analyses revealed that CTX induced splenic white pulp atrophy and an increase in the red: white pulp ratio, which was reversed by *A. ampeloprasum *Subsp* Iranicum* (Figure 7C and 7D).

## Discussion

Cyclophosphamide (CTX), a nitrogen mustard class anticancer drug, is utilized in the treatment of various cancer types. However, its usage is significantly constrained due to the induction of toxicity in non-cancerous cells. CTX exhibits the ability to impair DNA structure, eliminate immune cells, disrupt the proliferation and differentiation of B and T cells, and suppress both humoral and cellular immune responses. Administration of CTX results in myelosuppression and immunosuppression. Consequently, CTX has been employed in the establishment of immunosuppressed animal models across different research studies. Nevertheless, there exists considerable variation in the protocols and durations utilized in these studies aiming to induce CTX-induced immunosuppression (Haubitz, 2007; Shirani et al., 2015). In the current study, a murine model of CTX-induced immunosuppression was employed by intraperitoneally administration of CTX (50 mg/kg) from the beginning of the experiment for 5 days. The findings of the present study demonstrated that the mice treated with CTX showed significantly reduced body weight and immune organ indices, WBC count, spleen cell number, and lymphocyte proliferation ability, down-regulated cytokine (IFN-γ and IL-4,), and suppression of DTH and HA responses compared to the control group which indicated immunosuppression.

The *Allium* plants, which are widely distributed in the world, can increase host immune responses to various diseases by regulating immunity (Padiyappa et al., 2022). These findings have drawn considerable attention to investigating whether *A. ampeloprasum *Subsp* Iranicum *could be a promising natural immunomodulator. The present study showed that *A. ampeloprasum *Subsp* Iranicum *can restore several parameters including body and spleen weight, spleen cellularity, WBC counts, HA titer, DTH response, and lymphocyte proliferation ability, and cytokine production suggesting that *A. ampeloprasum *Subsp* Iranicum *can exert its protective effect in CTX-induced immunosuppressive mice. 

The result of this study indicated that exposure to CTX resulted in a lower mass of the spleen, which might be associated with an overall decreased spleen cell number because of the direct cytolytic action of CTX on lymphocytes. Treatment with *A. ampeloprasum *Subsp* Iranicum * led to a higher spleen weight, possibly because of the increase in the population of different cells in these organs (Figures 2 and 3). Our findings support the conclusions of Radjabian et al., 2019; *A. ampeloprasum *Subsp* Iranicum *had the highest impact on the proliferation of splenocytes compared to other plants (*A. sativum*, *A. elburzense*, and *A. asarense*).

Lymphocyte proliferation is a critical aspect of lymphocyte activation, which is considered a significant milestone in the initiation of adaptive immunity (Heinzel et al., 2018). In this study, it was observed that *A. ampeloprasum *Subsp* Iranicum *notably enhanced the proliferation of T lymphocytes induced by PHA and B-lymphocytes induced by LPS in CTX-treated mice with immunosuppression. This observation suggests that *A. ampeloprasum *Subsp* Iranicum* exerts its protective effects on CTX-induced immunosuppressive mice by enhancing both cellular and humoral immunity.

Immunoglobulins are the basic member of humoral immunity, which can activate complement, specifically bind to antigens, and display an important function includes neutralizing infectious agents, and preventing bacterial bound and/or entry into host cells (Schroeder and Cavacini, 2010). Consist with the results of lymphocyte proliferation, the HA test in the current study showed a considerable decrease in the production of antibodies against SRBC in the group that received CTX compared to the normal saline group, while *A. ampeloprasum *Subsp* Iranicum *extract was able to improve the reduction of antibody production caused by CTX (Figure 4).

T lymphocytes play a vital function in host defense against microbial pathogens through the production of a wide range of cytokines. Specific immune responses play a crucial role in the immune system, and they can be classified into two main types: Type 1 T helper (Th1) and Type 2 T helper (Th2) cell responses. These two types of immune responses have distinct functions and serve different purposes within the body. Th1 lymphocytes are associated with cellular immune responses such as delayed hypersensitivity by producing cytokines predominantly IFN-γ, whereas Th2 stimulates the humoral response, promotes B cell proliferation, and induces antibody production by secreting cytokines including IL-4 (Rajaei, 2022).

 According to Table 2, it was observed that *A. ampeloprasum *Subsp* Iranicum *effectively restored the levels of IFN-γ and IL-4 in immunosuppressed mice. This suggests that *A. ampeloprasum *Subsp* Iranicum* had a significant impact on activating Th1 and Th2 cells. DTH response assay is a valuable method for assessing the cellular immune response, which is triggered by direct stimulation of sensitive T cells upon contact with antigens. The outcomes of the DTH response demonstrated a strong immune response in mice treated with the hydroalcoholic extract of *A. ampeloprasum *Subsp* Iranicum*.

By measuring the DTH response, it is possible to indirectly assess the stimulation of Th2, Th1, and Th17 cells. It appears that *A. ampeloprasum *Subsp* Iranicum*, similar to other *Allium* species, has the potential to influence the balance of Th1/Th2 cells and even affect the activity of Th17 cells (Allen, 2013; Terhune et al., 2013).

Recent studies have delved into the fascinating realm of flavonoids and their impact on immune response. For instance, research by Farhadi et al. in 2014 highlighted how flavonoids can boost DTH and stimulate the secretion of IFNγ from Th1 cells (Farhadi et al., 2014). 

In the hydroalcoholic extract of *A. ampeloprasum *Subsp* Iranicum*, the flavonoid content was found to be quite significant, with a measure of 334.66 mg/g of dry extract based on quercetin. This observation provides strong evidence linking the observed strengthening effect to this particular compound. Moving on to another fascinating component, phytol, it has been discovered in various *Allium* species, including *A. ampeloprasum *Subsp* Iranicum*. Presently, phytol is being utilized as an adjuvant to enhance immune responses triggered by vaccines. Exciting research conducted by Aachoui et al. in 2011 and Harke et al. in 2021 show cases the safety and high efficacy of this combined approach in stimulating the immune system when used in conjunction with different vaccines (Aachoui et al., 2011; Harke et al., 2021).

Additionally, the organosulfur compounds found in *Allium* species play a crucial role in their medicinal effects. In the case of *A. ampeloprasum *Subsp* Iranicum*, the most abundant bioactive compound is dipropyl trisulfide, constituting approximately 34.77% of the total chemical compounds in the plant. Intriguingly, a study by Arsenijevic et al. in 2021 indicated that a solution containing dipropyl polysulfide compounds (with 8.48% dipropyl trisulfide) could influence the balance of Th17/Treg cells in favor of regulatory T (Treg) cells. This finding, as demonstrated by Aryakia and Karimi in 2018, has significant implications for immune regulation. These studies shed light on the remarkable potential of compounds found in *A. ampeloprasum *Subsp* Iranicum*, paving the way for further investigation into their immunostimulatory properties and possible applications in optimizing immune responses (Aryakia and Karimi, 2018).

The present study revealed that the hydroalcoholic extract of *A. ampeloprasum *Subsp* Iranicum* has the potential to enhance the recovery from immunosuppression induced by CTX. This is achieved by regulating cytokine secretion levels and strengthening the immune system. The protective effect of the *A. ampeloprasum *Subsp* Iranicum* extract on the immune system may be attributed to the presence of polyphenols, flavonoids, and sulfur compounds. Conducting further comprehensive studies is necessary to gain a better understanding of the impact of *A. ampeloprasum *Subsp* Iranicum* on various immune system mechanisms.

**Table 1:** Impacts of subacute orally exposure to *A. ampeloprasum *Subsp* Iranicum* for 14 days on white blood cell counts in cyclophosphamide-exposed mice

**CTX**	***A. iranicum*** **+CTX**	***A. iranicum***	**NS**	
1.85±0.79^**^	3.25±0.14^**++^	5.48±0.18^***+++^	2.44±1.08	**WBC (10** ^4^ **/µl)**
1.47±0.12^*^	2.20±0.21^*+^	4.10±0.97^***+++^	1.86±0.90	**LYM (10** ^4^ **/µl)**
0.20±0.14^*^	0.68±0.20^**+++^	0.97±0.21^*+^	0.38±0.13	**MONO (10** ^4^ **/µl)**
0.18±0.05	0.37±0.09^*+^	0.41±0.09^*+^	0.20±0.08	**NEU (10** ^4^ **/µl)**

Data are expressed as mean±SD. *p<0.05, **p<0.01 and ^***^p< 0.001: significant changes compared to the control group (NS). +p<0.05, ++p<0.01, and +++p<0.001 compared to CTX group. Monocytes (MONO; Neutrophils (NEU); Lymphocytes (LYM); White blood cell (WBC).

**Table 2:** Impacts of subacute orally exposure to *A. ampeloprasum *Subsp* Iranicum* for 14 days on cytokines concentrations in cyclophosphamide-exposed mice.

**CTX**	***A. ampeloprasum *** **Subsp** *** Iranicum*** **+ CTX**	***A. ampeloprasum *** **Subsp** *** Iranicum ***	**NS**	
175±15^***^	255±21^+++^	238±18^+++^	264±18	IFN-𝛾 (pg/ml)
86±19^***^	150±21^+++^	141±27^+++^	154± 23	IL-4 (pg/ml)

Data are presented as mean±SD. ^***^p<0.001: significant changes compared to the control group (NS). +++p<0.001 compared to CTX group. NS (Normal Saline), CTX (Cyclophosphamide), interferon 𝛾 (IFN-𝛾), interleukin-4 (IL-4)
